# A Double-Edged Sword: Volatile Anesthetic Effects on the Neonatal Brain

**DOI:** 10.3390/brainsci4020273

**Published:** 2014-04-16

**Authors:** Sunny Chiao, Zhiyi Zuo

**Affiliations:** Department of Anesthesiology, University of Virginia, Charlottesville, VA 22908, USA; E-Mail: ssc4r@virginia.edu

**Keywords:** anesthesia, anesthetics, neuroprotection, neurotoxicity, pediatrics, volatile anesthetics, preconditioning, postconditioning, ischemic conditioning

## Abstract

The use of volatile anesthetics, a group of general anesthetics, is an exceedingly common practice. These anesthetics may have neuroprotective effects. Over the last decade, anesthetic induced neurotoxicity in pediatric populations has gained a certain notoriety based on pre-clinical cell and animal studies demonstrating that general anesthetics may induce neurotoxicity, including neuroapoptosis, neurodegeneration, and long-term neurocognitive and behavioral deficits. With hundreds of millions of people having surgery under general anesthesia worldwide, and roughly six million children annually in the U.S. alone, the importance of clearly defining toxic or protective effects of general anesthetics cannot be overstated. Yet, with our expanding body of knowledge, we have come to learn that perhaps not all volatile anesthetics have the same pharmacological profiles; certain ones may have a more favorable neurotoxic profile and may actually exhibit neuroprotection in specific populations and situations. Thus far, very few clinical studies exist, and have not yet been convincing enough to alter our practice. This review will provide an update on current data regarding volatile anesthetic induced neurotoxicity and neuroprotection in neonatal and infant populations. In addition, this paper will discuss ongoing studies and the trajectory of further research over the coming years.

## 1. Introduction

Tens of millions of surgeries are performed every year in the US alone, approximately six million of which are on pediatric populations [1]. The development of general anesthetics has been a huge breakthrough in modern medicine; there is no argument that they have revolutionized and prolonged the quality and longevity of human life by enabling increasingly complex surgeries and procedures to take place. However, over recent years, the safety of these same anesthetic agents has come under scrutiny after the realization that immature animals demonstrate neurodegeneration, long-lasting neurocognitive and behavioral deficiencies, and elderly animals have learning and memory impairment after exposure to general anesthesia [2,3]. Animal studies have demonstrated that general anesthetics cause increased neuronal apoptosis and changes to the developing brain in animals [3,4,5,6,7,8,9,10,11,12,13]. In addition to these immediate changes, some pre-clinical studies also suggest that exposure to general anesthesia may be associated with changes in neurobehavior and neurocognition that potentially persist for many months [3,6,8,9,10,12,13,14,15]. Other studies have reached the opposite conclusion: exposure of specific populations to general anesthetics either have no long term adverse effects, or may actually have benefit and act as neuroprotective interventions [16,17,18,19,20].

Our current understanding is that anesthetic exposures in the middle stages of life have no serious (or at least measurable) long-term neurocognitive or neurodevelopmental effects. However, for those patients at the extremes of age, does volatile anesthetic exposure truly cause neuronal apoptosis, and does that have lasting consequences on their future neurocognitive functions?

This overview will briefly discuss proposed mechanisms of general anesthesia, the development of the human nervous system, and arguments for and against anesthetic-induced neuroprotection and neurotoxicity.

## 2. Methods

PubMed, Cinahl, and Medline searches were performed between October 2013 and January 2014 regarding anesthesia induced neuroprotection and neurotoxicity, with special focus on pediatric populations. Citations from reference lists of relevant articles, abstracts from the American Society of Anesthesiologists, International Anesthesia Research Society were also reviewed.

## 3. Results

There are numerous animal models of anesthesia-induced neuronal toxicity and subsequent neurobehavioral and cognitive disturbances. There are also a large number of animal studies on neuroprotection induced by exposure to volatile anesthetics. Several retrospective studies have identified neurological disturbances in young children after neonatal exposure to anesthesia and surgery. No prospective studies are available to evaluate neurocognitive and neurobehavioral outcomes in young children after neonatal exposure to volatile anesthetics.

## 4. Physiology of Brain Development, Synaptogenesis and Neuroapoptosis

The development of the human brain is a very complex process characterized by cell proliferation, migration, and differentiation, during which cells become specialized and distinct from one another. Environmental exposures or insults that interfere with any one of these processes can therefore have lasting impacts on neurodevelopment. This explosion of rapid central nervous system (CNS) maturation and development, or “brain growth spurt” in humans begins in utero and continues for the first 2–3 years of life [21]. Throughout this period, the brain and nervous system are particularly vulnerable to insults from the environment, drugs, hypoxia, hypoglycemia, or maybe exposure to anesthetic agents. Neurons begin to migrate from about six weeks gestational age *in utero* until about five months after birth, with myelination approximately halfway completed at six months in the corpus collosum, although maturation of various cell types does not form synchronously or even at the same rate, which explains why in animal models, disruptive exposures at different times result in different effects on the brain [22,23]. During this process, they establish physical contact among themselves and construct complex circuits [24,25].

Apoptosis is the process of programmed cell death. This is different from necrosis, which is cellular death as a result of cellular trauma or injury. Neuroapoptosis, or apoptosis in brain and neuronal tissue, is critical in the normal development and differentiation of the nervous system. Indeed, disturbances of programmed cell death lead to embryonic mortality or gross anatomical malformation [26]. Life begins with an actual excess of neurons, which are selectively trimmed away by programmed cell death, or apoptosis. This process is heavily influenced by environmental and external cues. Inter-neuronal synaptic connections and communication are crucial to maintaining and forming normal functional tissue, an effect that is conserved across species even as distant as *Caenorhabditis elegans* and *Drosophila melanogaster* [26].

Apoptosis occurs via two different mechanisms: the intrinsic (mitochondrial) pathway or the extrinsic (“death receptor”) pathway. The intrinsic pathway is caused by mitochondrial dysfunction, and subsequent cytochrome c and caspase-9 release from mitochondria. The extrinsic pathway involves the death induced signaling complex and caspase-8. Both intrinsic and extrinsic pathways result in a common pathway: activation of caspase-3 and subsequent cellular apoptosis [27].

## 5. Volatile Anesthetics—Mechanisms of Action

Volatile anesthetics are general anesthetic agents that are delivered by inhalation (in contrast to intravenously administered anesthetics) in combination with oxygen and air to induce general anesthesia, a reversible state of unconsciousness. In addition to loss of consciousness, general anesthesia also provides amnesia, analgesia, immobility, and control of autonomic responses during surgery. Volatile anesthetics have been shown to provide all of these components to some degree, take effect quickly, and demonstrate an ease of monitoring that have made them very popular. Especially in the pediatric age group, volatile anesthetics are an attractive option for induction of anesthesia and can minimize stress of the experience for the child.

Commonly used modern volatile anesthetics include desflurane, sevoflurane, and isoflurane, which are all halogenated hydrocarbons.

Desflurane (Suprane) was introduced in 1992, and has a low solubility in blood. This drug affords rapid onset and offset of action that allows for easy and quick titration of anesthetic dose to a surgical plane of anesthesia, as well as rapid emergence and recovery at the end of anesthesia. One study of pediatric patients (mean age: 5 years old) undergoing surgical anesthesia exhibited a mean time to eye opening on verbal command and extubation at 6.9 ± 3.8 min and 6.6 ± 3.0 min, respectively [28]. It has the negative attributes of requiring a complex vaporizer system for delivery, and being highly irritating to the airway. In fact, inhalational induction with desflurane resulted in airway complications in 30%–40% of healthy patients with occasional prolonged or even failed inductions [29]. This irritating effect can induce moderate to severe laryngospasm (49%) and moderate to severe coughing (58%) during desflurane induction of anesthesia in pediatric populations [30].

Sevoflurane (Ultane) was first synthesized in 1968, but was not used in clinical practice until the 1990s. It has a relatively pleasant smell and is not irritating to the airway [31]. It has a higher solubility as compared with desflurane, and therefore has a slower onset and offset of action, but is a good choice for an induction agent. Successful induction with 5% sevoflurane in O_2_-N_2_O mixture in a group of un-premedicated patients undergoing major gynecologic surgery has been shown to occur within 109 ± 25 s [32]. Time to recovery and eye opening on command after anesthesia were also relatively short at 7.5 ± 0.5 min [33]. This was slightly longer than desflurane in pediatric patients, with the sevoflurane group requiring 6.2 ± 2.7 min and 9.3 ± 3.7 min to eye opening on verbal command and extubation, respectively [28]. Sevoflurane has become a mainstay of inhalational induction of anesthesia, especially in pediatric populations. It is significantly more expensive than desflurane and isoflurane.

Isoflurane (Forane) has been used since the 1980s and is one of the more heavily studied volatile anesthetics. It is more potent than sevoflurane and desflurane, and has a slower onset and recovery time than both desflurane and sevoflurane. It is mildly pungent, making it a less practical induction agent [34].

Although it is widely accepted that volatile anesthetics exert their effects on the neuronal membrane of the central nervous system to alter synaptic transmission, the exact sites and mechanisms of action of these agents have eluded definitive characterization. Several theories exist, including the lipid solubility/anesthetic potency (Meyer-Overton) correlation, and the membrane protein hypothesis. The Meyer-Overton Correlation was independently described by Hans Meyer in 1899 and Charles Overton in 1901 [35,36]. Both identified a correlation that anesthetic potency is directly related to lipid solubility [36,37]. This correlation assumes that general anesthetics act by simple diffusion, or essentially dissolving, into the lipid rich portion of neurons. Therefore, by disrupting the activity of these lipid layers, general anesthetics induce anesthesia. While the Meyer-Overton Correlation was more or less accepted for many decades, it has come under scrutiny for having significant “exceptions to the rule”. For instance, enantiomers with the same solubility exert different anesthetic potencies, and non-immobilizers with similar chemical structures and lipid solubility do not exhibit general anesthetic properties [38,39]. Thus, the Meyer-Overton Correlation is no longer considered to be an appropriate theory to fully explain induction of general anesthesia by anesthetics.

More recently, an alternative mechanism of action has been proposed. In the 1980s, Franks and Lieb found that volatile anesthetics were able to exert inhibitory activity on cytochrome P450 and the firefly enzyme luciferase, even in the complete absence of lipids [37]. These inhibitory effects were directly correlated with anesthetic properties. This finding implies that volatile anesthetics exhibit selectivity to bind with specific targets, as opposed to simple disruption of lipid membranes to induce anesthesia.

The two neurotransmitters that have come to the forefront of research into the mechanisms of anesthesia are glutamate (an excitatory neurotransmitter) and γ-aminobutyric acid (GABA) (an inhibitory neurotransmitter) [40,41]. In simplistic terms, enhanced inhibition by an increase in GABA_A_ receptor activity, or decreased excitation by blockade of glutamate receptor activity is thought to induce general anesthesia [42,43,44]. Other protein targets that have since been implicated in this process include ion channels, neurotransmitter transporters, opioid receptors, muscarinic acetylcholine receptors, α_2_-adrenergic receptors, and 5-hydroxytryptophan (HT)_2a_ receptors [45,46,47]. It is likely that the effects of volatile anesthetics are primarily from agonist activity at the GABA_A_ receptor, but also may have action at multiple target sites including *N*-methyl-d-aspartate (NMDA), acetylcholine, glycine, and serotonin receptors. NMDA receptors are a subtype of glutamate receptors.

## 6. Volatile Anesthetic-Induced Neuroprotection

For several decades, it has been accepted that volatile anesthetics can be neuroprotective during periods of brain ischemia. This was first evidenced in the 1960s, when it was shown that patients undergoing carotid occlusion under cyclopropane anesthesia had greater tolerance to cerebral ischemia [48]. Further studies demonstrated that general anesthetics showed protective effects against cell injury process or stimulus, such as neuroapoptosis, degeneration, and ischemia, although most evidence has been in adult animals [48,49,50,51]. This neuroprotection can occur when anesthetics are applied during, before (preconditioning), or after (postconditioning) periods of ischemia.

Ischemic pre-conditioning refers to the effect that a short period of non-lethal ischemic exposure before a long period of ischemic exposure reduces cell injury. Pre-conditioning has two phases: (1) the acute phase, which begins minutes after the application of preconditioning stimulus and lasts for a few hours; and (2) the delayed phase, which does not begin until several hours after the initial pre-conditioning. The delayed phase may have several days of the effective time-window [52,53,54,55]. Ischemic postconditioning refers to protection induced by short episodes of ischemia during the stages of reperfusion after a long period of ischemia. Postconditioning may be more clinically applicable than pre-conditioning, as it is difficult to identify patients at risk for ischemic injury prior to the actual insult taking place. Application of volatile anesthesia during peri-ischemic periods appears to provide protection through a mechanism similar to ischemic conditioning [56,57,58].

### 6.1. Animal Studies

Neuroprotection induced by applying anesthetics during ischemic brain injury has been reported in multiple volatile agents, although current experimental data is mostly derived from rodent stroke models. Isoflurane-induced neuroprotection has been described in stroke models including after subarachnoid hemorrhage [59] or middle cerebral artery occlusion [60,61]. In some studies, isoflurane appears to have a dose dependent effect on protection that is also influenced by the severity and duration of brain ischemia [62]. Some data suggest that isoflurane has long-term effects on delay and reduction of injury, with effects described as persisting as long as 7 days or even up to 8 weeks after the ischemic event [60,63,64]. Sevoflurane induced neuroprotection during ischemia may be neuroprotective in cerebral ischemia for up to 28 days after ischemia [65].

Neuroprotection from the application of volatile anesthetics during periods of neurologic injury and ischemia has been demonstrated. It is a potentially useful tool during cardiopulmonary bypass, deep hypothermic circulatory arrest (DHCA), or other periods of brain ischemia. In pigs, administration of desflurane before and after DHCA has been shown to be protective against ischemia, as evidenced by less tissue damage and neuroapoptosis [66,67]. In other organ systems, anti-inflammatory effects have been reported in the heart, kidneys, and lung [68,69].

Neonatal rats exposed to isoflurane prior to induction of brain hypoxia and ischemia experienced preconditioning effects in the brain. In 7 day old rats, which are thought to have the same “brain developmental age” as developmentally immature infants, 1%–1.5% isoflurane was used to precondition 24 h prior to an induced episode of brain hypoxia and ischemia of unilateral cerebral hemisphere [18,70]. Seven days later, isoflurane preconditioned brain had less brain tissue loss than brain without isoflurane preconditioning. This response was abolished in those also treated with aminoguanidine, an inhibitor of NO synthase, suggesting that isoflurane induced neuroprotection is mediated by nitric oxide synthase [18]. This effect may also be mediated by increased expression of bcl-2 (an anti-apoptotic protein), or NMDA receptor activation [17,18,71]. In another study, 6 day old rats were preconditioned with 1.5% isoflurane for 30 min 24 h before induced brain hypoxia and ischemia. Motor coordination, learning, memory, and neuropathology were tested 1 month later. At 7 days after exposure, those that had received isoflurane pre-conditioning experienced brain mass decrease by 10% as compared with 20% in the untreated group, and also exhibited better motor coordination 1 month after exposure, implying lasting effects of isoflurane induced neuroprotection [17]. In addition, neuronal density decrease after hypoxia and ischemia was not as dramatic in the pre-treated group, although there does not appear to be a mortality benefit from exposing neonatal rats to isoflurane prior to ischemia [17,18]. As mentioned before, preconditioning may be a difficult effect to be tested in humans, as it is not always possible to predict when ischemia will occur.

Isoflurane exposure also decreased frequency of ischemic depolarizations, a possible contributor to ischemic damage, during focal cerebral ischemia. These isoflurane exposed rats had similarly decreased infarct volumes when compared with pentobarbital exposed rats [72]. Rats experience reduction in ischemic brain injury after postconditioning with isoflurane. In a study of adult rats, isoflurane exposure reduced brain infarct volumes and apoptotic cells and neurological deficits at four weeks after ischemic insult by middle cerebral arterial occlusion, an effect possibly mediated by inhibition of inflammatory cytokine IL-1β [68]. This was also seen in a model of ischemia from oxygen-glucose deprivation [20]. A study of neonatal rat pups also supports the potential therapeutic value of postconditioning [73]. Rats postconditioned with isoflurane after intracerebral hemorrhage experienced reduction in brain edema, apoptotic cell death, and neurobehavioral deficits [74,75].

**Figure 1:**
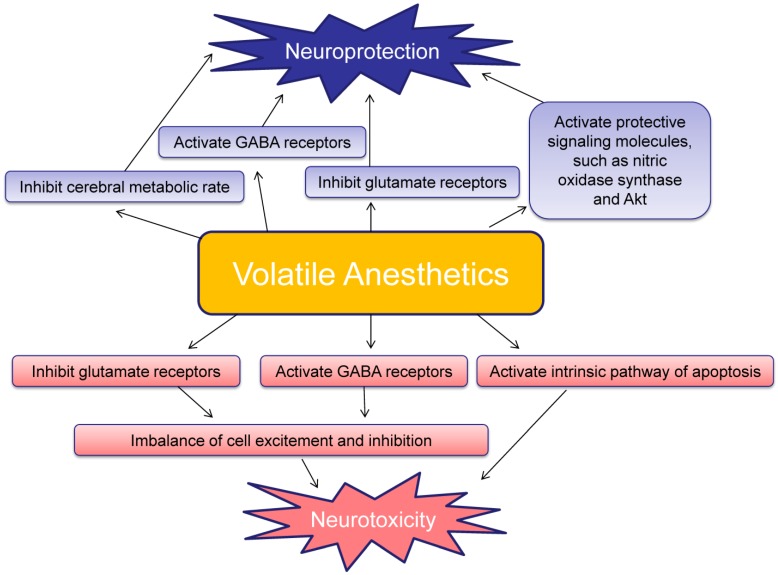
Schematic of the neuroprotective and neurotoxic effects induced by volatile anesthetics.

Volatile anesthetics-induced protective effect may be explained by a variety of hypotheses. General anesthesia decreases the metabolic rate of the brain, and thereby reduces oxygen consumption and minimizes hypoxia. Some models have also described inhibition of glutamate NMDA receptor over-activation, activation of mitochondrial adenosine-5′-triphosphate sensitive potassium channels, and preservation of calcium/calmodulin dependent protein kinase II (CaMKII) levels as possible mechanisms of anesthetic induced neuroprotection [19,20]. The protective effects of preconditioning and postconditioning may be additive possibly due to the different mechanisms that are activated [19,20] (Figure 1).

### 6.2. Clinical Studies

Most evidence for neuroprotection induced by volatile anesthetics is from adult populations. Multiple volatile agents have been linked to neuroprotection in adults. Desflurane has demonstrated enhanced tissue oxygenation and attenuated reduction in tissue pO2, and inhibition of lactic acidosis and decrease in pH during craniotomy [76]. During carotid endarterectomy, a reduction in critical cerebral blood flow and ischemic changes as seen on electroencephalography (EEG) was greater with exposure to isoflurane than halothane or enflurane, two older volatile anesthetics [77]. However, when comparing isoflurane anesthesia with intravenous brain protective anesthetics (propofol, etomidate, pentobarbital) for intracranial aneurysm surgery, patients tolerated longer duration of temporary iatrogenic MCA occlusion with the latter strategy [78].

While a small handful of studies have examined anesthetic protection in pediatric populations, they have thus far been centered on the myocardial protection, with one study demonstrating that preconditioning decreased release of CK-MB post-operatively after ventricular septal defect repair [79,80]. No studies specifically addressing neuroprotection induced by volatile anesthetics in pediatric populations have been published to date. Nevertheless, many clinical situations, especially during the perioperative period, may benefit from volatile anesthetic preconditioning- and postconditioning-induced neuroprotection if this protection is approved in humans including pediatric patients. For example, brain ischemia may occur during the surgery to correct congenital heart diseases. The time for these ischemic events can be reasonably accurately predicted.

## 7. Volatile Anesthetic-Induced Neurotoxicity

### 7.1. Animal Studies

After knowing that inhibiting NMDA receptors or activating GABA receptors can induce cell apoptosis in the neonatal rat brain [4], a study in 2003 described structural and long-term functional deficits in rats after prolonged exposure to a combination of isoflurane, nitrous oxide, and midazolam [3]. This finding has been reproducible in several studies [7,9,10,13,81,82]. A small study (*n* = 5) of neonatal non-human primate rhesus macaque brains after a fairly prolonged surgical plane of isoflurane (5 h) but without surgical stimulation demonstrated a 13-fold increase in neuroapoptosis 3 h after exposure [7]. Neonatal rats exposed to sevoflurane experienced short-term memory impairment [83].

Patterns of apoptosis are not uniformly scattered, but are more highly concentrated in certain areas including the primary visual cortex, temporal/somatosensory cortices, and the frontal cortex. Apoptosis of neurons seemed to be heavily more influenced by their geographic location than by their cell types; pyramidal neurons, interneurons, and multipolar neurons are all similarly affected [7]. Histologically, there is a notable decrease of hippocampal stem cell pool, neurogenesis, and cell density [9]. Neurobehavioral impairment does not necessarily correlate with neuropathological change or death of neurons [6].

Simultaneous potentiation of GABA_A_ receptors and antagonism of NMDA receptors may unfortunately have a synergistic effect in potentiating neuroapoptosis as well as altering long-term behaviors; this is particularly concerning when one considers the not infrequent practice of mixing various anesthetic agents together in order to provide a balanced anesthetic [3,11,81]. For instance, exposure to isoflurane alone demonstrated dose dependent neurodegeneration, but this was increased in exposure to both isoflurane and midazolam. Exposure to three different agents (N_2_O, midazolam, and isoflurane) resulted in even more severe neurodegeneration in terms of both neuronal loss and damage [3,8]. Resultant learning and memory disabilities persisted into adolescence and adulthood in animal models [3].

The effects of volatile anesthetics may be dependent upon the timing of exposure. When comparing seven-day-old rodents and sixty-day-old rodents exposed to isoflurane, researchers demonstrated significant impairment in learning and recognition in young but not adult animals [9,13]. However, one study concluded that the detrimental effects of volatile anesthetic exposure in young animals do not appear to persist into adulthood [9].

With regards to studies comparing neurotoxicity of currently used anesthetics, the data are also conflicting. A comparison of exposure to six hours of 0.6 minimum alveolar concentration of desflurane, isoflurane, and sevoflurane in neonatal mice exhibited significant, and similar, neurodegeneration in the superficial neocortex [84]. Another comparison of the same three volatile anesthetics at approximately 0.75 mean alveolar concentration at three or six hours showed greater levels of desflurane induced neuroapoptosis and impaired working memory than from sevoflurane or isoflurane [85]. A third comparison of sevoflurane and isoflurane exposure of seven day old rats to six hours of 0.5 minimum alveolar concentration demonstrated increased apoptosis from both agents, although isoflurane exposure had greater neurodegeneration [86].

Plenty of preclinical evidence exists in the form of demonstrable neurotoxicity of volatile anesthetics on animals. While awaiting the results of further clinical studies, we should exercise caution when extrapolating animal data to humans. Most studies exist in the rat or mouse, whose brains and even lifespans are vastly different from humans. Studies on non-human primates would be more applicable to our population in question. Currently, Ansgar Brambrink at Oregon Health & Science University is studying the long-term outcome of single *versus* triple exposure of infant non-human primates to anesthesia. His work is supported by the IARS Frontiers in Anesthesia Research Award [87]. The FDA’s National Center for Toxicological Research (NCTR) is conducting non-clinical studies in rodents and non-human primates to further characterize mechanisms of neurotoxicity of general anesthetics, long term cognitive deficits related to these exposures, strategies to minimize adverse anesthetic effects, and the utility of PET imaging to monitor neurotoxicity associated with anesthesia [87].

### 7.2. Suggested Mechanisms of Neuroapoptosis Induced by General Anesthesia

Glutamate and GABA are the major excitatory and inhibitory neurotransmitters, respectively. Glutamate, which has action on NMDA receptors, has trophic functions in the developing brain; it encourages cell growth, as well as cell migration. During the brain growth spurt period, NMDA receptor bearing neurons are highly reliant on glutamate and are at risk for degeneration [70,88,89]. Neonatal rats exposed to even a single dose of an NMDA antagonist experienced widespread apoptotic neurodegeneration in a manner resembling programmed cell death, with the highest effects seen at younger ages. In humans, peak NMDA receptor expression is during weeks 20–22 of gestation. Therefore, exposure of the developing fetus or neonate to volatile anesthetics can be considered high risk for neuroapoptosis and neurodegeneration [4]. Neonatal neurons may be dependent upon NMDA receptor stimulation; withdrawal or inhibition of NMDA receptor stimulation therefore triggers apoptosis [4] (Figure 1).

GABA is typically an inhibitory neurotransmitter. However, in the immature nervous system, it paradoxically also has excitatory properties. GABA heavily influences brain development, differentiation, growth rate, and synapse formation. Under normal circumstances, GABA receptor activation triggers action potentials and influx of calcium into the intracellular space. With potentiation of GABA receptors, the receptor may become over-activated and experience an overwhelming influx of calcium and result in apoptosis [90,91,92] (Figure 1).

A clinical example of these effects is neonatal alcohol exposure, which has been known for several decades to adversely affect the neonatal brain. In rat studies, ethanol exposure induces apoptosis and neurodegeneration in a dose and time-dependent fashion consistent with the pattern of NMDA antagonism and GABA_A_ agonist [8,81,93]. The combination of a GABA agonist and NMDA antagonist has been shown to cause widespread neurodegeneration in neonatal animals [3,11]. Fetal exposure to ethanol has also been associated with a variety of behavioral and psychiatric disturbances that persist into adulthood, including hyperactivity and attention deficit hyperactivity disorder (ADHD), learning disabilities, mental retardation, psychosis, and major depressive disorder [93]. Multiple areas of suspected vulnerability in the brain have been identified and linked with cognitive and behavioral impairment [3,6,7,8,9,10,94].

### 7.3. Clinical Studies

Clinical evidence for or against anesthetic induced neurotoxicity in humans is very weak. A few studies have shown that multiple (but not one) exposures to general anesthesia due to surgery carry an increased risk of development of learning disability [95,96,97,98]. Multiple studies have produced results that demonstrate no association between early anesthetic exposure/surgery and the development of cognitive or behavioral disturbances [99,100,101,102]. Since children in all of these studies had surgery, it is difficult to know whether any effects on cognition or behavior are due to surgery or an anesthetic.

One study described an increased risk in language and cognition deficits after only one anesthetic exposure, although this was not a consistent finding throughout all of the cognitive and behavioral testing [103]. Importantly, the study best suited to control for confounders, a twin study of unexposed and exposed children, did not show an association between general anesthetic exposure for surgery and the development of learning disability [100].

In 2009, Wilder studied a population based retrospective birth cohort from Olmsted County, Minnesota in search of an association between anesthetic exposure before 4 years old and the development of reading, writing, math, and learning disabilities. Of the 5357 children enrolled, 593 received anesthesia before age 4. In this study, a single exposure of anesthesia was not associated with an increased risk of learning disability (HR 1.0; 95% CI: 0.79–1.27), but multiple exposures to anesthesia were. A history of two anesthetic exposures had a hazard ratio of 1.59 (95% CI: 1.06–2.37) and a history of three or more exposures carried an even greater hazard ratio of 2.6 (95% CI: 1.60–4.24). This study did not differentiate between specific anesthetic agents, although 87.9% of patients received halothane (a volatile anesthetic no longer used in the USA. but may be used in other countries), and 90.7% received nitrous oxide [95].

In the same year, DiMaggio *et al*. [96] conducted a retrospective cohort analysis of 383 enrollees of the New York State Medicaid program to examine the association of anesthesia for inguinal hernia surgery with the incidence of behavioral and developmental disorders. These subjects all underwent hernia surgery during the first 3 years of life. The exposed group was compared with a sample of 5050 age-matched children without any history of hernia repair. They found that 4.4% of children with history of hernia repair and anesthetic exposure were later diagnosed with a behavioral or developmental disorder, as compared to 1.2% in the control group, equivalent to an adjusted hazard ratio of 2.3 (95% CI: 1.3, 4.1) [96].

The same group later also retrospectively studied a sibling birth cohort to minimize environmental confounders. They enrolled 10,450 siblings, 304 of which were exposed to surgery and anesthesia before age 3. The 304 exposed children and 10,146 unexposed children were followed up until diagnosed with a developmental or behavioral disorder, were lost to follow up, or the end of 2005. The hazard ratio of developmental or behavioral disorders was greater with increased number of surgeries and anesthetic exposures. The estimated hazard ratio of developmental or behavioral disorders associated with any exposure to anesthesia prior to age 3 years was 1.6 (95% CI: 1.4, 1.8). The risk increased from 1.1 (95% CI: 0.8, 1.4) for 1 operation to 2.9 (94% CI: 2.5, 3.1) for 2 operations and 4.0 (95% CI: 3.5, 4.5) for ≥3 operations. The incidence of developmental or behavioral disorders was 128.2 diagnoses per 1000 person-years for exposed individuals, and 56.3 diagnoses per 1000 person-years for unexposed individuals. However, the relative risk in a matched analysis of 138 sibling pairs was 0.9 (95% CI: 0.6, 1.4) [97].

A retrospective pilot study was conducted by Kalkman *et al.* in 2009 to assess neurobehavioral development in children aged 12.5–15.8 years old who had received anesthesia for a pediatric urological procedure before age six. This study was largely inconclusive. Although it concluded that children receiving surgery before age 2 showed more behavioral disturbances, the results were not statistically significant. This pilot study enrolled 314 children; an estimated minimum enrollment of 2268 was suggested to definitively confirm or refute anesthesia effects [99].

In 2012, Ing, *et al.* [103] analyzed 2608 children from the Western Australian Pregnancy Cohort (Raine) Study, which included 2868 children born from 1989 to 1992. Of the 2608 children, 321 had history of anesthesia exposure before age 3. They were followed up at age 10 at which point outcomes in language, cognitive function, motor skills, and behavior were tested with a battery of tests. On average, children with history of anesthetic exposure had lower scores than unexposed peers in receptive and expressive language, as demonstrated by testing with Clinical Evaluation of Language Fundamentals (CELF) Receptive (CELF-R) and Expressive (CELF-E), and Colored Progressive Matrices (CPM). Exposed individuals demonstrated language disabilities (CELF-R: adjusted risk ratio (aRR), 1.87; 95% CI, 1.20–2.93, CELF-E: aRR, 1.72; 95% CI, 1.12–2.64), and cognition (CPM: aRR, 1.69; 95% CI, 1.13–2.53). An increased aRR for disability in language and cognition persisted *even with a single exposure to anesthesia* (CELF-R aRR, 2.41; 95% CI, 1.40–4.17, and CPM aRR, 1.73; 95% CI, 1.04–2.88). No differences were noted between groups with another receptive language assessment tool, the Peabody Picture Vocabulary (PPVT) test, or with regards to tested behavior and motor function [103].

Flick *et al.* [98] in 2011 arranged a matched cohort study of 8548 children from Rochester, MN. The exposed group consisted of 350 children who were exposed to anesthesia before age two, and these were matched to 700 unexposed controls. They were followed up at age 19 and tested for learning disability, speech-language, behavioral, or emotional disorders. They reported findings that exposure to multiple anesthetics was associated with an increased occurrence of individualized education programs for speech or language in the exposed group, but found no association with individualized education programs for emotional or behavioral reasons. In addition, they found that a history of multiple exposures to general anesthesia had a hazard ratio of 2.12 risk of developing a learning disability. A single exposure to anesthesia was not associated with an increased risk of a learning disability. The majority of these patients had also received a combination of halothane and nitrous oxide [98].

Hansen *et al.* [102] compared academic performances of all children who had undergone inguinal hernia repair in infancy with random, age-matched samples. They examined average test scores when subjects were in the 9th grade. Again, the exposure group performed worse than the control, but these results were not statistically significant. They found no evidence that a single, brief anesthetic exposure was correlated with worse academic performance at age 15 or 16 [102]. In a group of Danish birth cohorts undergoing surgery for pyloric stenosis before three months of age, Hansen *et al.* [101] also found no statistically significant difference between the two groups with regards to academic performance.

Bartels *et al.* [100] sought to answer a question that has long stymied researchers: does early anesthetic exposure cause cognitive and behavioral deficits, or is this correlation seen because sick children need surgery, and are they more predisposed to illness, surgery, and learning disabilities to begin with? They identified 1143 monozygotic twin pairs and assessed for a history of anesthetic exposure before age three. The subjects were then divided into concordant exposed, concordant non-exposed, and discordant exposure groups, and assessed around age 12 for educational achievement and cognitive problems based on standardized tests and teacher ratings. Twins exposed to anesthesia before age 3 had significantly lower achievement scores and higher incidences of cognitive problems than unexposed twins. Importantly, discordant twin pairs (one sibling exposed to anesthesia before age three and one sibling unexposed before age three) did not differ in achievement or cognition. This study was therefore did not demonstrate *causality* and may rather point to a predilection of susceptible individuals to needing surgical intervention and development of cognitive deficits [100].

The above studies have reached conflicting conclusions. It is still unclear whether there is association with early exposure to general anesthetics and later development of learning disability or behavioral disturbances. Nevertheless, the existing evidence suggests that anesthetic effect on learning and memory after an early exposure in humans is small, if there is any.

## 8. Looking Forward on Anesthetic-Induced Neurotoxicity Research

There are currently several studies in various stages of completion that may help to shed light and deepen our understanding of the impact of anesthetics on the brain of pediatric populations.

The U.S. Food and Drug Administration (FDA) has taken efforts to address the safety of anesthetics in pediatric populations. In 2007, committee members from the FDA’s Anesthetic and Life Support Drugs Advisory Committee reached a consensus to defer anesthesia for elective procedures in children less than 3 years of age, although they agreed that evidence was insufficient to change the current practice of anesthesia. The FDA has also entered into a partnership with the International Anesthesia Research Society (IARS) called SmartTots (Strategies for Mitigating Anesthesia-Related Neuro-Toxicity in Tots) As of the 2011 IARS *SmartTots* session, two goals have been defined: to improve our understanding of anesthetic-induced neurotoxicity at a cellular level, and to improve our understanding of anesthesia-related neurobehavioral sequelae in mammalian species exposed to anesthesia during critical stages of brain development [87,104].

The PANDA (Pediatric Anesthesia and NeuroDevelopment Assessment) Study, led by Sun and colleagues will perform a prospective assessment of neuropsychological functions in a retrospective cohort of children. The exposed cohort will consist of children undergoing general anesthesia for inguinal hernia repair before age 36 months, and the unexposed cohort will consist of their siblings without any exposure before age 36 months. The subjects will undergo behavioral and cognitive testing with the Wechsler Abbreviated Scale of Intelligence (WASI), NEuroPSYchological Assessment, second edition (NEPSY II), Child Behavior Checklist, age 6–18 (CBCL 6–18) and Conners’ Parent Rating Scales, 3rd Edition. They have already performed a pilot feasibility study of 28 exposed-unexposed sibling pairs at age 6–11 years old with follow-up testing an average of 7 years later, which preliminarily demonstrated no differences in average scores between the two groups [105,106].

The General Anesthesia Spinal (GAS) study is an international study group of 26 institutions led by Andrew Davidson, Neil Morton, and Mary Ellen McCann with support from the FDA and U.S. National Institutes of Health (NIH). It is a randomized trial of approximately 700 infants younger than 60 weeks undergoing hernia repair that are randomized to either general anesthesia with sevoflurane or regional spinal anesthesia. They will be followed-up for five years, with testing at ages 2 and 5 years. At age two, they will be tested with the Bayley Scales for Infant Development-III, and at age 5, will undergo testing with the Wechsler Preschool and Primary Scale of Intelligence-III in addition to other tests. This study is expected to conclude in 2017 [107].

Another similar pending study is a prospective randomized clinical trial headed by de Graaff in the Netherlands. It will compare general and regional spinal anesthesia for hernia repair. Neurospychological testing and evaluation will be performed in 2018 when patients reach age 5 [108].

The Mayo Clinic, in collaboration with the FDA’s National Center for Toxicological Research (NCTR), is conducting the MASK (Mayo Safety in Kids) Study. It is a population-based cohort study of long term cognitive development in children from Rochester, Minnesota. They will compare children with no exposure, a single exposure, or multiple exposures to general anesthesia prior to age three and assess cognitive performance using a battery of neurocognitive tests, including the operant test battery [87].

Tom Hansen’s group from the Netherlands is conducting a birth cohort study to test academic performance in adolescent patients who received general anesthesia for inguinal hernia repair as infants [87].

It can hardly be argued that we should minimize both the length and number of exposures to anesthetics. In the interest of protecting the immature brain against untoward effects of volatile anesthetics, should we then withhold these potentially toxic drugs? In a 2012 editorial, Australian author Davidson invites us to reexamine the current belief that all neonates need a hypnotic component to anesthesia [109].

“Regardless of whether or not sevoflurane causes any clinically relevant toxicity, is it time to question the mantra that all babies need a hypnotic agent such as sevoflurane?” [109].

Only a few decades ago, anesthesia for neonates consisted of only nitrous oxide, oxygen, and paralytics, ironically out of concern for untoward side effects of anesthetic agents. In the 1980s, several landmark studies demonstrated that this practice was inadequate and was in fact associated with increased stress response, hemodynamic instability and poorer outcomes [110,111,112,113,114]; anesthetic techniques were accordingly changed. We now know that analgesia is absolutely critical to prevent altered pain processing, development of chronic pain disorders, and behavioral and cognitive dysfunction in adulthood [113,115,116]. A 1992 study comparing anesthetic techniques for neonatal cardiac surgery found that deep anesthesia with high dose sufentanil was associated with statistically significant reductions in stress response as compared with a group who received lighter anesthesia with halothane and morphine [112]. If adequate analgesia is sufficient to provide sedation, maintain hemodynamic stability, and minimize humoral and inflammatory responses, do these patients benefit from the addition of general anesthetic?

## 9. Conclusions

Volatile anesthetics are indispensable tools that are used on tens of millions of patients each year. They are particularly useful in pediatric anesthesia as a means to induce anesthesia for sedation or surgery in a relatively non-stressful or traumatic fashion in this immature age group. Although we do not have a complete understanding of the mechanism of action(s) that induce(s) anesthesia, it may be through effects on proteins. We now have considerable *in vitro* and animal data implicating all currently used general anesthetics, including volatile anesthetics, in inducing neurotoxicity. These changes are dependent on dose and long duration of anesthetic exposure. On the other hand, strong evidence also exists for the neuroprotective effects of volatile anesthesia against brain ischemia and various detrimental insults. These effects usually require short and clinically relevant exposure times. Nevertheless, we should exercise caution when trying to apply results from animal and *in vitro* studies to our practice.

The neuroprotective and neurotoxic effects of volatile anesthetics in humans are not clear. No studies have been performed to determined volatile anesthetic induced neuroprotection in children. Clinical data thus far has remained too weak to either support or refute claims of anesthetic mediated neurotoxicity in children. No changes can be recommended to the current practice of anesthesia, or with regards to selection of superiority of one anesthetic agent over another. It may be reasonable to delay completely elective pediatric surgery until at least four years of age. Several clinical trials are currently ongoing that may provide pivotal insight into this question.
